# Effect of nutritional intervention combined with vitamin D on glucose metabolism, sex hormones and inflammatory factors in patients with polycystic ovary syndrome

**DOI:** 10.5937/jomb0-53948

**Published:** 2025-06-13

**Authors:** Xinqin Kang, Lin Liu

**Affiliations:** 1 Zigong Third People's Hospital, Department of Obstetrics and Gynecology, Zigong, Sichuang, China; 2 Zigong Third People's Hospital, Department of Orthopedics, Zigong, Sichuang, China

**Keywords:** endocrine, inflammatory response, nutritional interventions, nutritional status, polycystic ovary syndrome, endokrini, inflamatorni odgovor, nutritivne intervencije, nutritivni status, sindrom policističnih jajnika

## Abstract

**Background:**

Polycystic ovary syndrome is a very common endocrine and metabolic disease in clinical practice. Most polycystic ovary syndrome patients are complicated with obesity, a condition associated with an elevated risk of long-term complications such as diabetes, hypertension, and endometrial cancer, seriously threatening the health of patients. The best way to treat this disease is to use drugs to promote ovulation, adjust the menstrual period, and restore pregnancy. However, the drugs used to treat obesity and polycystic ovary syndrome usually have entirely opposite effects, resulting in unsatisfactory rehabilitation.

**Methods:**

This study selected 102 obese or overweight PCOS patients treated in our hospital from July 2022 to July 2024 as the research subjects. Sex hormones: The levels of prolactin (PRL), testosterone (T), follicle-stimulating hormone (FSH), and luteinising hormone (LH) were measured by electrochemiluminescence. Inflammation: C-reactive protein (CRP), white blood cell count (WBC), and procalcitonin (PCT) were examined by an automated blood cell counter. 46 (control group) of the 102 were treated with a conventional treatment scheme, and the other 56 (experimental group) were intervened by nutritional intervention combined with vitamin D based on conventional treatment.

**Results:**

Both groups showed reductions in sex hormone levels after treatment, with the PRL, T, LH, and FSH in the experimental group being (5.12±0.51) ng/mL, (1.22±0.32) ng/mL, (9.14±1.61) mIU/mL, and (5.01±0.42) mIU/mL, respectively, all of which were lower compared with the control group (P<0.05).

**Conclusions:**

In this study, we intervened obese polycystic ovary syndrome patients with nutritional or conventional intervention and found that compared with conventional treatment, the posture, blood glucose metabolism, and lipid function of the patients who received nutritional intervention were more significantly metabolised, and the levels of inflammatory factors were more effectively inhibited, indicating the high clinical application value of nutritional intervention in obese polycystic ovary syndrome.

## Abstract (sr)

**Uvod:**

Sindrom policističnih jajnika veoma je česta endokrina i metabolička bolest u kliničkoj praksi. Većina pacijentkinja sa sindromom policističnih jajnika ima i gojaznost, stanje povezano s povećanim rizikom od dugoročnih komplikacija kao što su dijabetes, hipertenzija i rak endometrijuma, što ozbiljno ugrožava zdravlje pacijentkinja. Najbolji način za lečenje ovog sindroma jeste upotreba lekova za podsticanje ovulacije, regulisanje menstrualnog ciklusa i obnavljanje plodnosti. Međutim, lekovi koji se koriste za lečenje gojaznosti i sindroma policističnih jajnika obično imaju potpuno suprotne efekte, što dovodi do nezadovoljavajuće rehabilitacije.

**Metode:**

Ova studija je odabrala 102 gojazna ili prekomerno gojazna pacijenta sa PCOS-om koji su lečeni u našoj bolnici od jula 2022. do jula 2024. kao subjekte istraživanja. Seksualni hormoni: Nivoi prolaktina (PRL), testosterona (T), folikulostimulirajućeg hormona (FSH) i luteinizirajućeg hormona (LH) su mereni metodom elektrohemiluminiscencije. Upala: C-reaktivni protein (CRP), broj leukocita (WBC) i prokalcitonin (PCT) su ispitani pomoću automatskog brojača krvnih ćelija. Od ukupno 102 pacijentkinje, 46 (kontrolna grupa) lečeno je konvencionalnom terapijskom šemom, dok je kod preostalih 56 (eksperimentalna grupa) intervenirana je nutritivnom intervencijom u kombinaciji s vitaminom D na temelju konvencionalnog lečenja.

**Rezultati:**

Obe grupe su pokazale smanjenje nivoa seksualnih hormona nakon terapije, pri čemu su vrednosti PRL, T , LH i FSH u eksperimentalnoj grupi iznosile (5,12±0,51) ng/mL, (1,22±0,32) ng/mL, (9,14±1,61) mIU/mL i (5,01±0,42) mIU/mL, respektivno, što je bilo niže u poređenju sa kontrolnom grupom (P<0,05).

**Zaključak:**

U ovoj studiji smo intervenisali kod pacijentkinja sa gojaznošću i sindromom policističnih jajnika primenom nutritivne ili konvencionalne intervencije i utvrdili da, u poređenju sa konvencionalnim tretmanom, držanje tela, metabolizam glukoze i funkcija lipida kod pacijentkinja koje su primile nutritivnu intervenciju pokazuju značajnije poboljšanje, a nivoi inflamatornih faktora su efikasnije inhibirani. Ovo ukazuje na visok klinički značaj nutritivne intervencije u tretmanu sindroma policističnih jajnika u kom binaciji sa gojaznošću.

## Introduction

Polycystic ovary syndrome (PCOS), a commonly seen reproductive endocrine and metabolic disease with a predilection for women of childbearing age, is clinically manifested as excessive androgen or characterised by clinical or biochemical manifestations, persistent anovulation, and polycystic changes in the ovaries (e.g., irregular menstrual cycles, infertility, hirsutism, or acne) [1]. The exact cause of PCOS is currently unclear, and clinical evidence suggests that it may be related to genetic and environmental factors [2]. Obesity, mainly central obesity (or abdominal obesity), is a common manifestation of PCOS patients. Obese PCOS patients are more likely to experience long-term complications such as diabetes, hypertension, and endometrial cancer than normal people, seriously affecting their quality of life and health [3].

Reviewing previous data, we found that most studies believe that with weight loss, some patients can develop spontaneous ovulation, regular menstruation, and improved insulin resistance and blood lipid metabolism, so weight loss is the preferred treatment for obese PCOS patients [4]. Nutritional intervention is an important adjuvant therapy for obese PCOS patients, which can further improve the clinical metabolic and endocrine indexes of obese PCOS patients [5].

Based on this, this paper conducts a detailed analysis of the effect of nutritional intervention on the endocrine function and nutritional status of obese PCOS patients, highlighting the importance of nutritional intervention in enhancing the quality of obese PCOS patients. These results may provide a more comprehensive reference and guidance for future clinical use in the treatment of PCOS.

## Materials and methods

### Study subjects

We used G*power software to calculate the sample size needed for this study. This study selected 102 obese PCOS patients treated in our hospital from July 2022 to July 2024 as the research subjects for retrospective analysis. Inclusion criteria: The included patients all aged above 20 and met the diagnostic criteria for PCOS [6] obesity or overweight (body mass index [BMI]≥24 kg/m^2^), with insulin resistance and complete clinical data; patients and their family members were informed and provided written informed consent forms. We obtained consent from the patients and their family. Exclusion criteria: The excluded patients were those complicated with serious primary diseases of the liver and/or kidneys, cardio-cerebrovascular diseases, mental illness, or severe gastrointestinal diseases, those who had recently taken weight-loss-related drugs or endocrine-regulating drugs, and those could not insist on treatment but quit, withdrew, or lost to follow up. 46 (control group) of the 102 were treated with conventional treatment scheme, and the other 56 (experimental group) were intervened by nutritional intervention combined with vitamin D based on conventional treatment. Note: This study was conducted after obtaining approval from the Ethics Committee of our hospital (No. 2022202).

### Methods

Control group: Ethinylestradiol and Cyproterone Acetate Tablets (1 tablet/dose, 21d consecutively considered as one cycle) were taken orally starting from the 5th day of the menstrual cycle for 3 consecutive cycles, with one week interval between each cycle. Experimental group: Vitamin D was added to the control group. The nursing staff gave dietary guidance to patients according to their weight, body fat, illness, etc.: patients were told to ensure a balanced and reasonable diet for three meals a day, with more protein-rich food and fresh fruits and vegetables and controlled intake of carbohydrates and fat. In addition, they were encouraged to develop eating habits of regular quantitative meals and reasonable intake of various meals. The nursing staff kept detailed records of the patient’s daily diet. Besides, medical staff gained patients’ trust based on respecting patients, worked together with patients to identify the causes of obesity, evaluated patients’ physical quality and clinical manifestations, and formulated reasonable nutritional interventions from the aspects of diet, exercise, psychology, etc. according to pa tients’ different needs. First, the nursing staff encouraged patients to eat low-energy foods rich in protein and fibre and low in fat, salt, and sugar, developed recipes (three meals a day) and made diet records. Second, real-time monitoring of patient indicators was carried out, and dietary adjustments were made according to specific conditions. Third, patients were required to complete 3–5 times of moderate-intensity sessions per week, with each session lasting at least 30 minutes. Fourth, the relevant information was regularly publicised to patients and explained in combination with the characteristics of symptoms, emphasising the role of nutrition and exercise intervention, increasing patients’ self-confidence, promoting patients’ self-awareness, and urging and encouraging patients to insist on completing the intervention plan.

### Sample collection and testing

Fasting venous blood was collected in the early morning before and after the intervention to determine the levels of serum fasting blood glucose (FPG), fasting insulin (FINS), Total cholesterol (TC), total triglyceride (TG), as well as low- (LDL-C), high-density lipoprotein cholesterol (HDL-C), Albumin (ALB) and Total protein (TP) with an automatic biochemical analyser (BS-800, Myers, USA), and the Homeostasis model assessment of insulin resistance (HOMA-IR) index was calculated. The levels of prolactin (PRL), testosterone (T), follicle-stimulating hormone (FSH), and luteinising hormone (LH) were measured by electrochemiluminescence (CL-2600i, Myers, USA). [5] Inflammation: C-reactive protein (CRP), white blood cell count (WBC), and procalcitonin (PCT) were examined by an automated blood cell counter (BC-5700, Myriad, USA).

### Outcome measures

(1) Nutritional status: The height, weight, waist circumference, ALB, TP, and hip circumference of patients were measured before and after the intervention, and the BMI and waist-hip ratio (WHR) were calculated and ALB, TP. (2) Blood glucose metabolism: FPG, FINS, and HOMA-IR. (3) Lipid function: TC, TG, LDL-C, and HDL-C. (4) Sex hormones: PRL, T, FSH, and LH. (5) Inflammation: CRP, WBC, and PCT.

### Statistical methods

Statistical analysis was conducted on the data results using SPSS 25.0 statistical software. Count data [n (%)] was compared using the chi-square test. Use the Shapiro-Wilk test to confirm the normal distribution of the data. Data that conformed to normal distribution were recorded as (x̄±s), and independent samples t-tests were used for between-group comparisons and paired t-tests for within-group comparisons. Data that did not conform to a normal distribution were recorded as median (interquartile spacing). Comparisons between groups were made using the nonparametric Mann-Whitney U test, and comparisons within groups were made using the Willcoxon rank sum test. P<0.05 was considered statistically significant.

## Results

### The two groups showed similar baseline data

The two groups’ ages, pregnancy history, marital status, and other baseline data were compared, and no significant difference was identified (P>0.05). Comparison of nutrient proteins, on the other hand, showed that ALB and TP were elevated in both groups after treatment. Still, the experimental group was higher than the control group (P<0.05), Table 1.

**Table 1:** Baseline information table.

Group	n	Age	First pregnancy Yes/No	Cigarette smoking No/Yes
Control group	46	28.9±4.4	27(58.7)/19(41.3)	43(93.5)/3(6.5)
Experimental group	56	28.4±3.3	35(62.5)/21(37.5)	51(91.1)/5(8.9)
* χ^2^/t *		0.655	0.153	0.202
* P *		0.514	0.695	0.653

The nutritional status of the experimental group after treatment was better than that of the control group.

**Figure 1:**
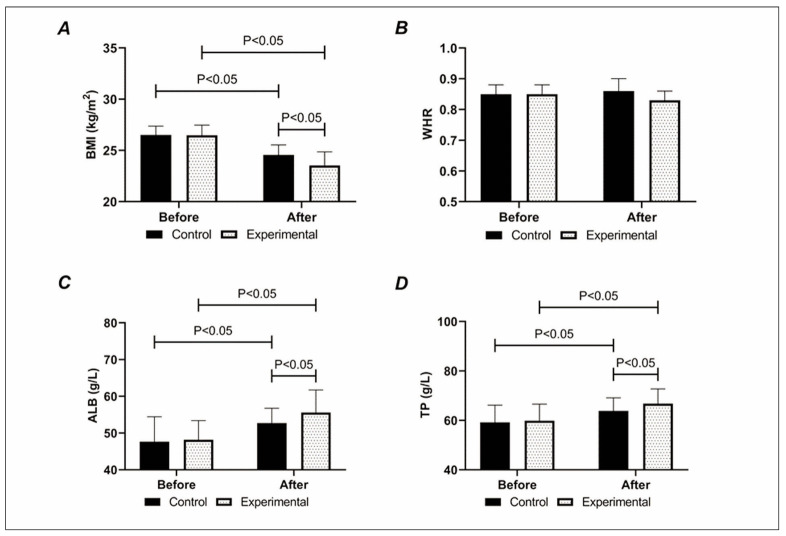
Comparison of (A) BMI, (B) WHR, (C) ALB, and (D) TP. The experimental group’s post-treatment BMI was lower than that of the control group, while ALB and TP were higher than that of the control group.

The two groups did not differ much in pre-treatment BMI and WHR (P>0.05). After treatment, WHR changed little in both groups (P>0.05); however, BMI decreased and was lower in the experimental group compared with the control group after treatment (P<0.05), Figure 1.

The blood glucose metabolism was better in the experimental group than in the control group after treatment.

**Figure 2:**
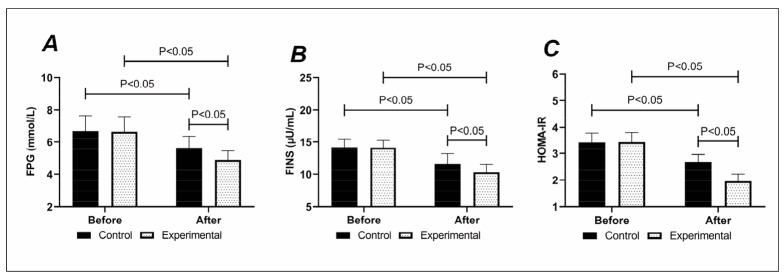
Comparison of (A) FPG, (B) FINS, and (C) HOMA-IR. FPG, FINS and HOMA-IR were lower in the experimental group than in the control group after treatment.

The two groups were also not statistically different in pre-treatment FPG, FINS, and HOMA-IR levels (P>0.05). FPG, FINS, and HOMA-IR were reduced in both groups after treatment, and the FPG, FINS, and HOMA-IR of the experimental group was lower compared with the control group (P<0.05), Figure 2.

The experimental group showed better lipid function than the control group after treatment.

**Figure 3:**
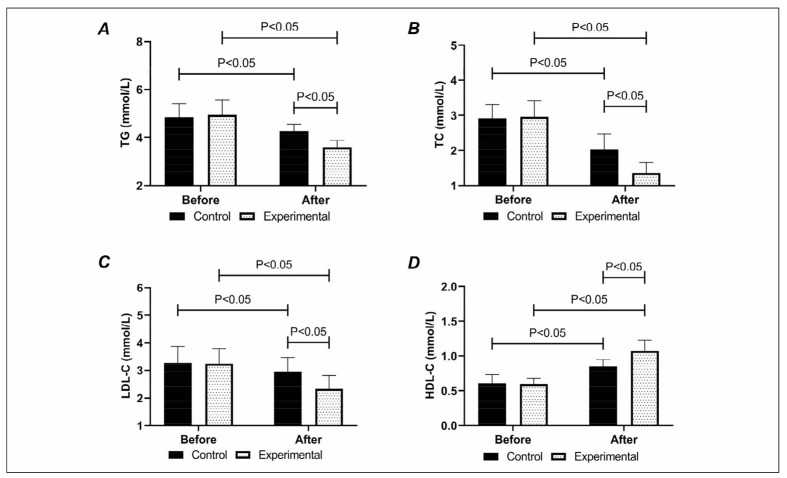
Comparison of (A) TG, (B) TC, (C) LDL-C, and (D) HDL-C. TG, TC and LDL-C in the experimental group were lower than those in the control group after treatment, while HDL-C was higher than those in the control group.

No significant inter-group difference was found in lipid function before treatment (P>0.05). After treatment, TG, TC, and LDL-C in both groups decreased, with more significant reductions in the experimental group (P<0.05); HDL-C increased and was higher in the experimental group compared with the control group (P<0.05), Figure 3.

The sex hormones were lower in the experimental group compared with the control group after treatment.

**Figure 4:**
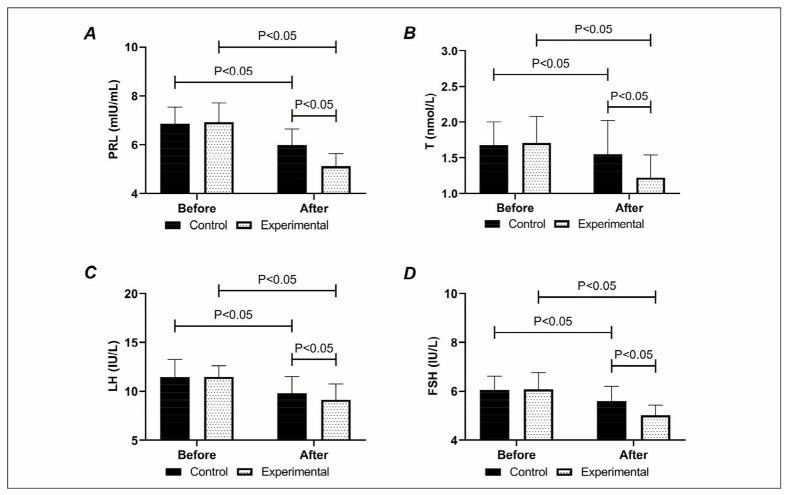
Comparison of (A) PRL, (B) T, (C) LH, and (D) FSH. PRL, T, LH, and FSH were lower in the experimental group than in the control group after treatment.

Both groups showed reductions in sex hormone levels after treatment, with the PRL, T, LH, and FSH in the experimental group being (5.12±0.51) ng/mL, (1.22±0.32) ng/mL, (9.14±1.61) mIU/mL, and (5.01±0.42) mIU/mL, respectively, all of which were lower compared with the control group (P<0.05), Figure 4.

Milder inflammation was determined in the experimental group compared with the control group after treatment.

**Figure 5:**
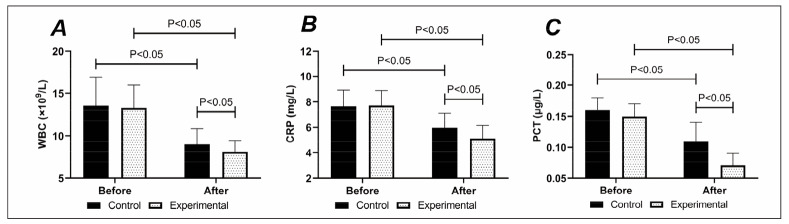
(A) Comparison of (A) WBC, (B) CRP. And (C) PCT. WBC, CRP and PCT were lower in the experimental group than in the control group after treatment.

Before treatment, there was no difference in CRP, WBC, and PCT between the two groups (P>0.05). CRP, WBC, and PCT were reduced in both groups after treatment, especially in the experimental group (P<0.05), Figure 5. 

## Discussion

This study found that nutritional intervention combined with vitamin D effectively improved the endocrine function and nutritional status of obese PCOS patients to provide a more reliable safety guarantee for their rehabilitation, which is worthy of clinical popularisation.

As we all know, PCOS is a common endocrine disorder and metabolic disease in clinical practice, with the vast majority of patients experiencing severe obesity symptoms and insulin resistance [7] [8]. The best treatment for this disease is to use medication to promote ovulation, adjust menstruation, and restore pregnancy [9]. However, the recovery of patients is not ideal, as drugs for obesity and PCOS usually have opposite effects. In this study, we found that the experimental group treated with nutritional intervention had a more significant decrease in BMI, FPG, FINS and HOMA-IR compared to the control group, suggesting that nutritional intervention is more effective in improving posture in obese PCOS patients. We speculate that this is mainly due to the nutritional intervention model, which reduces the intake of refined and added sugars with the help of dietary structure optimisation, improves insulin sensitivity, and effectively regulates metabolic rate, thus facilitating patients to obtain ideal treatment expectations. Most scholars have recognised nutritional intervention in clinical applications because of its advantages, such as being cost-effective and healthy [10] [11] [12]. Obese PCOS patients can effectively achieve rapid weight loss under the influence of a low-fat, high-protein diet, reduce the risk of organ dysfunction caused by excessive accumulation of organ fat, and alleviate the pathological progression of PCOS. This can be confirmed by the lower TC, TG, and LDL-C and higher HDL-C in the experimental group when comparing the lipid function between the two groups. Meanwhile, nutritional intervention enriches the diet structure and sets a clear target weight to further cooperate with patients to achieve the desired treatment level.

In the subsequent comparison of sex hormones, the lower post-treatment PRL, T, LH, and FSH in the experimental group versus the control group also suggest that nutritional intervention has a better effect on improving the endocrine function of obese PCOS, consistent with the research results of Che X et al. [13]. Research has shown that patients with obese PCOS often have increased androgen secretion, which inhibits follicular maturation, leading to follicular atresia and failure to form dominant follicles [14]. The nutritional intervention model, on the other hand, can effectively relieve the block of ovarian follicular maturation, promote follicular ovulation and maturation, enhance the metabolic level of patients, improve therapeutic effectiveness, and promote pregnancy [15] [16]. Furthermore, nutritional intervention is an economical, healthy, and convenient approach in clinical practice, with extremely high clinical applicability, which can provide a reliable guarantee for the prognosis of various PCOS patients to a great extent.

Finally, in comparing inflammatory reactions, the experimental group’s CRP, WBC, and PCT after treatment were lower than those of the control group, indicating a lower inflammatory response. Inflammatory factors, as one of the important members of the human immune system, are not only responsible for activating the innate and acquired immune systems and eliminating the invaders during the invasion of pathogens but can also mitigate inflammation and restore the body to the normal immune and physiological level after the elimination of the invaders [17]. For PCOS, the exacerbation of inflammatory responses is a typical manifestation and a key link in its pathological progression. Inflammatory factors have a significant regulatory effect on tissue differentiation and growth, and their elevated levels often lead to a decrease in the FSH sensitivity of follicles in the body, hindering follicle development, preventing follicle maturation, and resulting in anovulation [18]. The decrease in inflammatory factors in the experimental group under nutritional intervention is presumed to be due to the low carbohydrate and calories, which promote hepatic glycogenolysis, accelerate gluconeogenesis, and help patients lose weight. Weight control can effectively accelerate the recovery of insulin sensitivity, lower the level of synthetic androgens in the body, reduce the adverse factors affecting follicular development, decrease the levels of inflammatory factors in the body, promote ovulation rate, and increase the chance of conception.

However, this study also has many limitations. For example, the number of cases involved is small, and the follow-up period is short, which may bias the study results. In addition, more objective clinical indicators are needed to comprehensively evaluate nutritional intervention’s impact on PCOS. In the followup, we need to constantly optimise the specific details of nutritional intervention to provide a more reliable reference for clinical practice.

## Conclusion

Nutritional intervention can effectively enhance the endocrine function and nutritional status of obese PCOS patients and promote their physical health. Nutritional intervention has high clinical applicability as an economical, healthy, and convenient approach. It can provide a reliable and safe guarantee for the prognosis of PCOS patients to a great extent.

## Dodatak

### Availability of data and materials

Original data in this study are available from the corresponding author upon reasonable request.

### Funding

Not applicable.

### Author contributions

Xinqin Kang designed the study, wrote and revised the manuscript, and Lin Liu collected and analysed data. All authors read and approved the final submitted manuscript.

### Conflict of interest statement

All the authors declare that they have no conflict of interest in this work.
